# The complete chloroplast genome sequence of *Bolbitis angustipinna* (Hayata) H. Ito (Dryopteridaceae)

**DOI:** 10.1080/23802359.2025.2582543

**Published:** 2025-11-12

**Authors:** Lin Luo, Sai Wang, Huajing Zhou, Yuhan Fang, Tuantuan Wang, Huilong Ou, Jia Xie, Faguo Wang

**Affiliations:** ^a^ State Key Laboratory of Plant Diversity and Specialty Crops, South China Botanical Garden, Chinese Academy of Sciences, Guangzhou, China; ^b^ State Key Laboratory of Marine Resource Utilization in South China Sea, Hainan University, Haikou, China; ^c^ School of Ecology, Hainan University, Haikou, China; ^d^ School of Marine Biology and Fisheries, Hainan University, Haikou, China

**Keywords:** Chloroplast genome, *Bolbitis angustipinna*, *Bolbitis*, fern

## Abstract

*Bolbitis angustipinna* (Hayata) H. Ito is a rare ornamental fern primarily distributed throughout East Asia and Southeast Asia. Its complete chloroplast genome was assembled, and a phylogenetic tree was constructed using 15 fern species. The genome is 152,562 base pairs, with a large single-copy region (82,712 bp), a small single-copy region (21,403 bp), and two inverted repeat regions (24,208 bp). It encodes 115 genes, including 72 protein-coding genes, 35 tRNA genes, and 8 rRNA genes. Phylogenetic analysis shows that *B. angustipinna* is most closely related to *B. deltigera*, providing valuable genetic and evolutionary insights.

## Introduction

The Dryopteridaceae is a prominent fern family, with species that play critical roles in ecology, phylogeny, and conservation biology (Zhang et al. 2013; Liu et al. 2016). The genus *Bolbitis*, which belongs to this family, comprises approximately 80 species (Moran et al. 2010; PPGI 2016). *Bolbitis angustipinna* (Hayata) H. Ito (1938), a representative species of this genus, typically grows along streams in dense forests, and on rocks in tropical and subtropical areas (Dong and Zhang. 2005). Known for its abundant lateral pinnae (15–20 pairs), *B. angustipinna* forms an aesthetically pleasing plant shape, making it a popular choice for landscape design and garden decoration.

In addition to its ornamental value, studies on the embryonic development of *B. angustipinna* have highlighted the significance of its sexual generation, with observation of morphological features such as genitals and sperm providing valuable insights into the phylogenetic relationships within *Bolbitis* (Zhao et al. 2014). Despite this, there have been few reports on the chloroplast genome of *B. angustipinna*, and such genomic data could offer critical insights into its classification, evolutionary relationships, and adaptability.

Notably, *B. angustipinna* is listed as a vulnerable (VU) species in the ‘China Biodiversity Red List-Higher Plants Volume’ (Ministry of Environmental Protection of the People's Republic of China 2013), and is highly sensitive to the environment, which underscores the importance of its conservation and the need for further research. This study aims to sequence, assemble, and analyze the complete chloroplast genome of *B. angustipinna*, focusing on its genome structure, the characteristics of its chloroplast genome’s coding and non-coding regions, and its potential implications for plant phylogeny and horticultural applications. The results also contribute valuable reference material for the conservation of this species and other biodiversity efforts.

## Materials and methods

**Figure 1.**
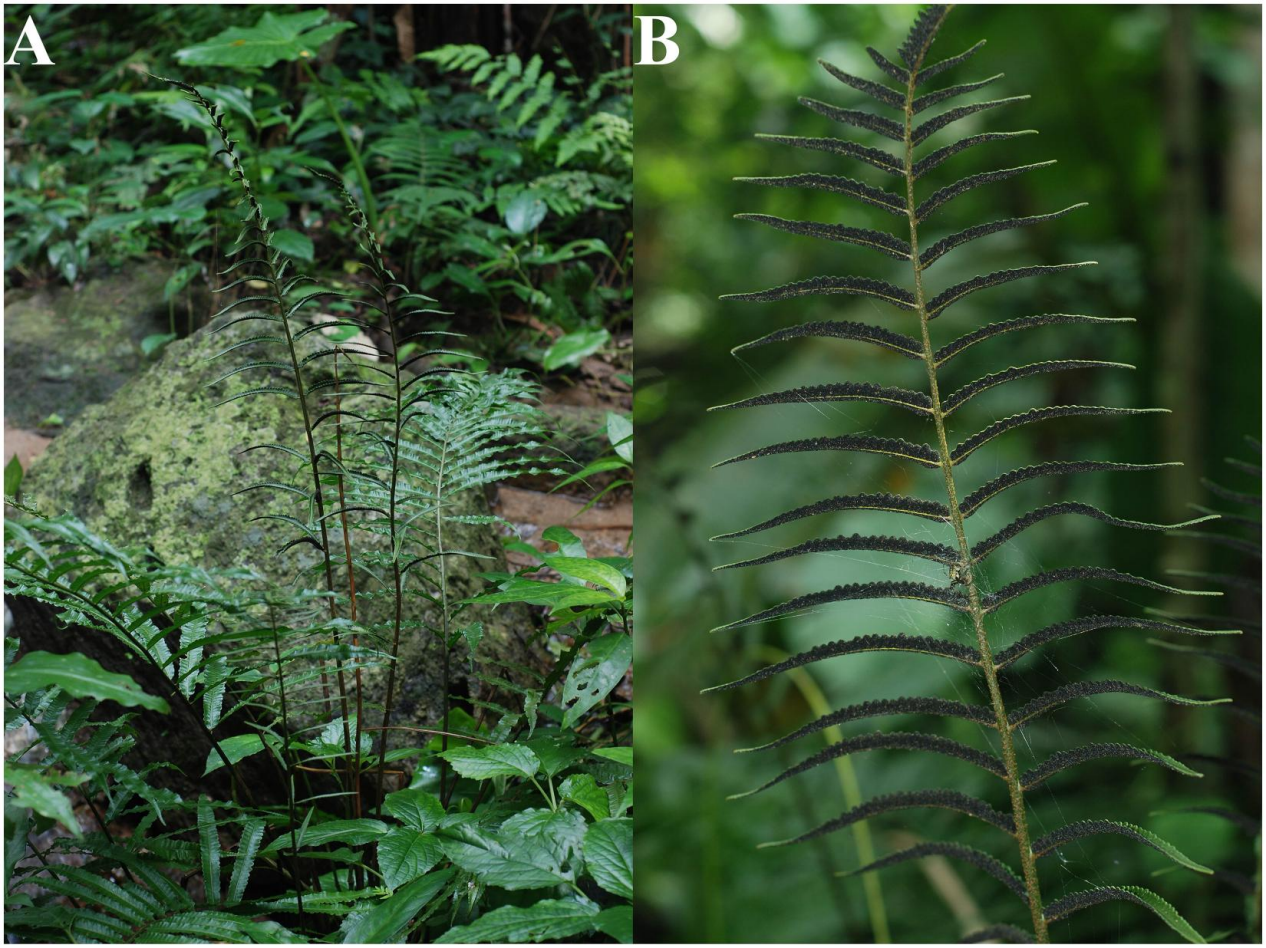
Species reference image of *B. angustipinna*. (A) Plant shape of *B. angustipinna*. (B) Morphological characteristics offertile fronds of *B. angustipinna.* The plant’s sterile fronds are elliptic and pinnate. The terminal pinna is linear-lanceolate with a subterminal bulbil. The lateral pinnae are alternate and linear-lanceolate. Sporangia inserted usually throughout abaxial surface, arrangement fully acrostichoid, sometimes with a sterile strip along costa. The species photo was taken by Faguo Wang in Limu Mountain in Qiongzhong County, Hainan Province, without any copyright issues.

Fresh leaves of *B. angustipinna* were collected from Limu Mountain in Qiongzhong County, Hainan Province (109.70674281°N, 18.70850097°E; altitude: 670 m). The plant specimens are stored in the herbarium of the South China Botanical Garden (http://herbarium.scbg.cas.cn/, Feiyan Zeng, zengfeiy@scbg.ac.cn), with specimen number WFG5608-1 (Figure 1(A,B)). Fresh plant samples were stored in liquid nitrogen for preservation. Genomic DNA was extracted from the leaf tissues using the CTAB method (Doyle 1987) and sequenced on the Illumina HiSeq X-Ten platform (Illumina Inc., San Diego, CA).

The resulting raw sequences were assembled using the GetOrganelle 1.7.5 toolkit, which relies on SPAdes 3.11 and Bowtie2 for sequence assembly and alignment (Langmead and Salzberg 2012; Bankevich et al. 2012; Jin et al. 2020). The genome coverage depth was determined using SAMtools v1.21 (Li et al. 2009), while the sequencing depth and coverage map were generated using ggplot2 (Ito and Murphy 2013) in R (Figure S1), which provided a view of the sequencing efficiency. The complete chloroplast genome was annotated and visualized using the CpGAVAS2 online tool (Shi et al. 2019) and CPGView (Shi et al. 2019), respectively, and the annotation was manually verified and refined in Geneious 9.0.2. The final annotated chloroplast genome was used to generate a circular diagram via Chloroplot (Zheng et al. 2020). The complete chloroplast genome sequence has been submitted to GenBank under accession number PP471972.

**Figure 2.**
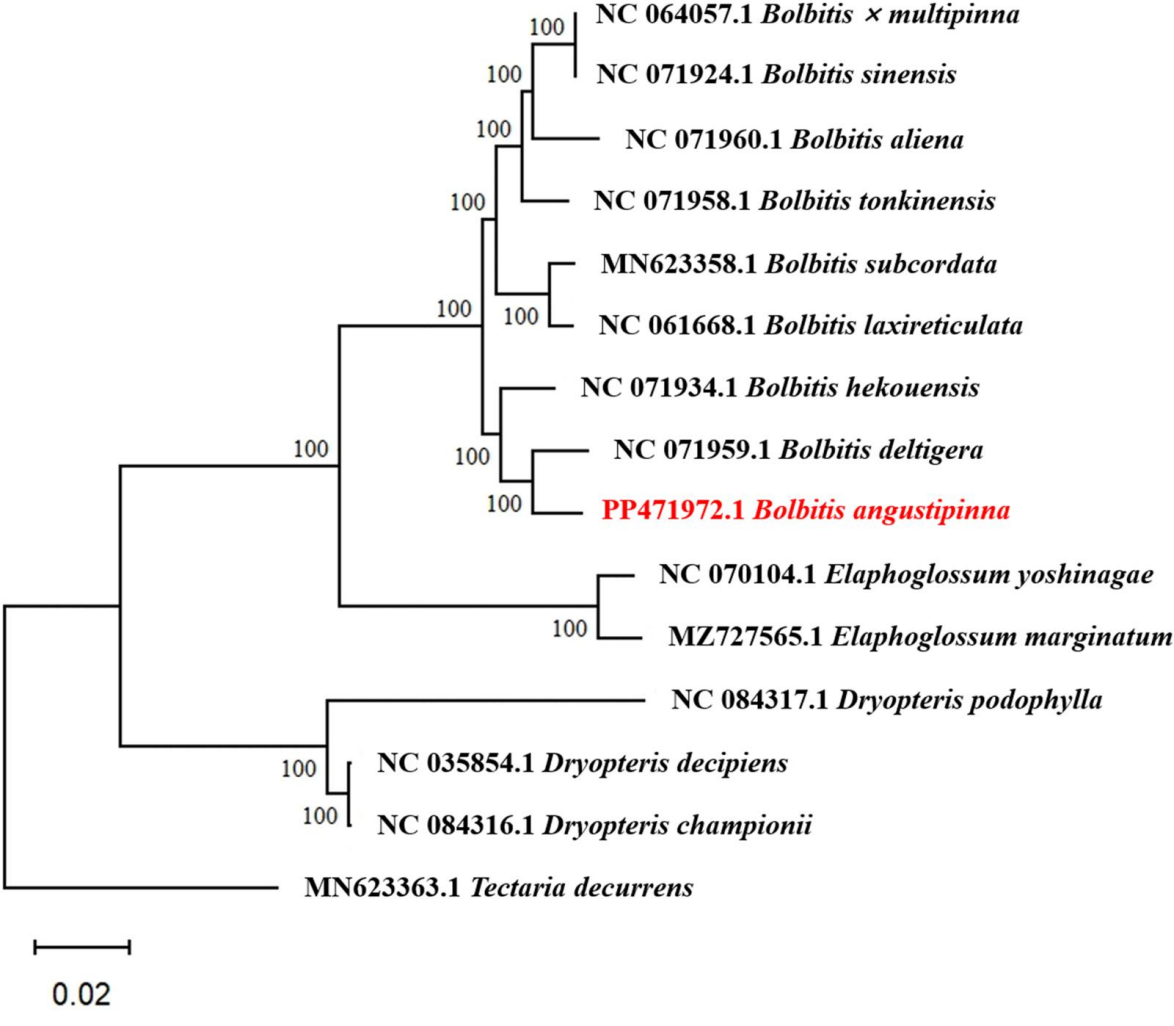
Maximum-likelihood and Bayesian phylogeny of *B. angustipinna* and related taxa based on 15 complete chloroplast genomes, with *Tectaria decurrens* from the Aspidiaceae family as the outgroup. Bootstrap values are indicated at each node. The target genome of *Bolbitis angustipinna* is shown in red. The following sequences were used: *Bolbitis sinensis* (NC071924), *Bolbitis x multipinna* (NC064057), *Bolbitis aliena* (NC071960), *Bolbitis tonkinensis* (NC071958), *Bolbitis laxireticulata* (NC061668), *Bolbitis subcordata* (MN623358) (Liu et al. 2020), *Bolbitis hekouensis* (NC071934), *Bolbitis deltigera* (NC071959), *Elaphoglossum marginatum* (MZ727565), *Elaphoglossum yoshinagae* (NC070104), *Dryopteris podophylla* (NC084317), *Dryopteris championii* (NC084316), *Dryopteris decipiens* (NC035854), and *Tectaria decurrens* (MN623363) (Liu et al. 2020).

To determine the phylogenetic position of *B. angustipinna*, we first conducted a BLAST search of its chloroplast genome against the NCBI nucleotide database (Altschul et al. 1990) to gather related sequences for constructing a phylogenetic tree (Figure 2). Chloroplast DNA sequences were aligned using MAFFT v.7 (Katoh and Standley 2013). These alignments were concatenated and analyzed using maximum-likelihood (ML) and Bayesian inference (BI) methods in RAxML v.7 (Stamatakis 2014) and MrBayes v3.2.1 (Ronquist et al. 2012), respectively, to generate the phylogenetic tree.

## Results

The raw sequencing data for *B. angustipinna* amounted to about 6 Gb. The complete chloroplast genome is 152,562 base pairs (bp) in length, and the assembled genome achieved an average sequencing depth of 339.45×, with a minimum depth of 10× and a maximum depth of 609× (Figure S1). The genome comprises a large single-copy (LSC) region of 82,712 bp, a small single-copy (SSC) region of 21,434 bp, and a pair of inverted repeat regions (IRa and IRb) of 24,208 bp. The total GC content of the chloroplast genome is 42%, with the highest GC content observed in the IR regions (45.2%), followed by the LSC region (41.1%), and the lowest GC content in the SSC region (38.4%).

**Figure 3.**
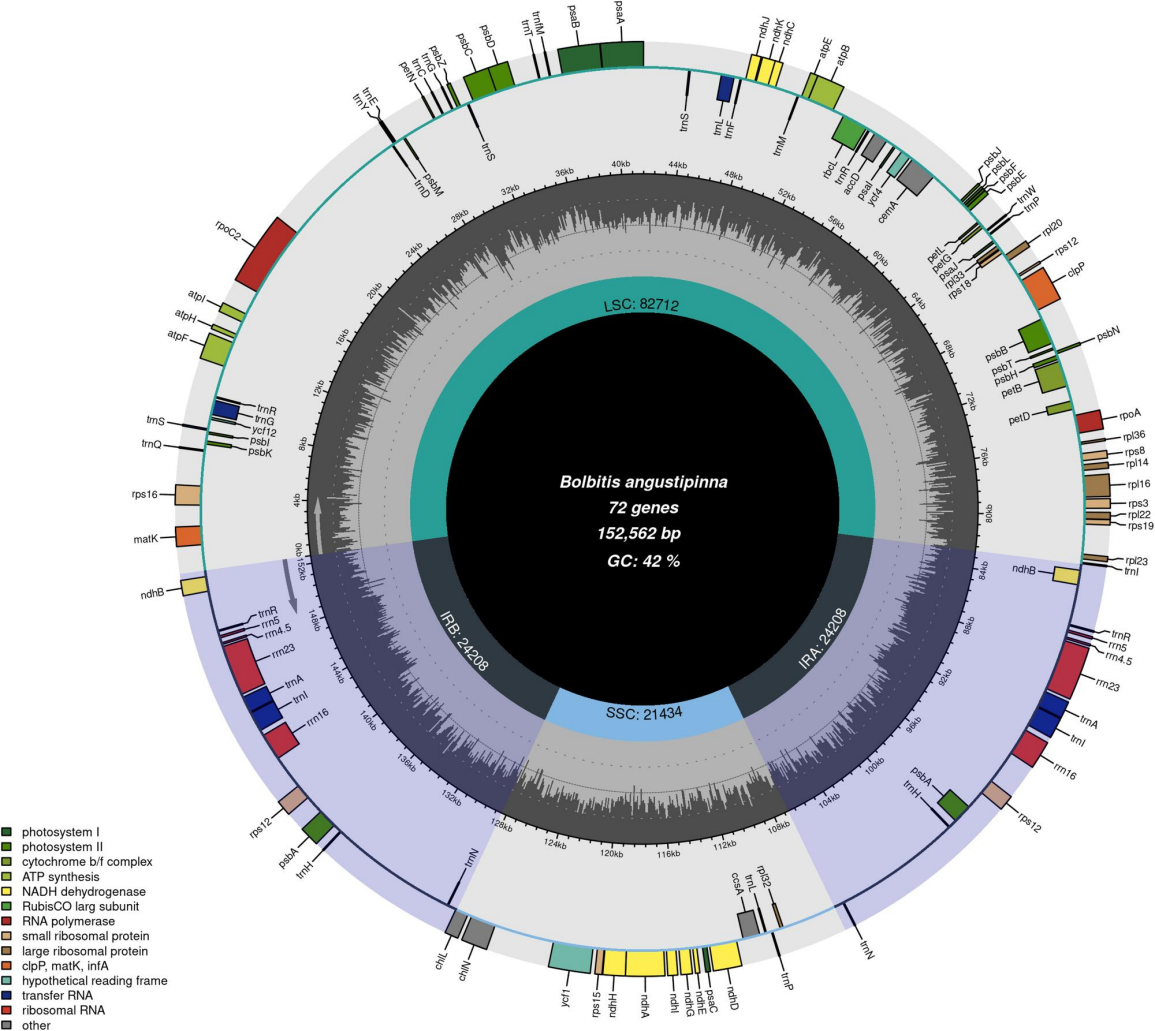
Gene map of chloroplast genome of *B. angustipinna* (PP471972). In the outermost circle, genes are represented by colored blocks of varying sizes. The intermediate circle illustrates the variation in GC content across different genomic regions. Arrows positioned on the inner and outer sides of this circle indicate genes transcribed in clockwise and counterclockwise directions, respectively. The innermost circle highlights the quadripartite structure – comprising the large single-copy (LSC), small single-copy (SSC), and inverted repeat regions (IRa and IRb) – using distinct colors to demarcate their respective boundaries, with lengths indicated.

The chloroplast genome encodes 115 genes, including 72 protein-coding genes, 35 tRNA genes, and 8 rRNA genes. Among these, 12 genes are duplicated, including 4 rRNA-encoding genes (*rrn4.5S*, *rrn5S*, *rrn16S*, *rrn23S*), 5 tRNA-encoding genes (*trnA-UGC*, *trnI-GAU*, *trnH-GUG*, *trnR-ACG*, *trnN-GUU*), and 3 protein-coding genes (*rps12*, *psaI*, *psbA*) (Figure 3). Furthermore, 6 cis-spliced genes (*rps16*, *atpF*, *clpP*, *petB*, *rpl16*, *ndhA*) and the trans-spliced gene *rps12* were identified in the chloroplast genome (Figures S2 and S3).

Phylogenetic analysis revealed that *B. angustipinna* is most closely related to *B. deltigera*, with strong support [ML bootstrap support (MLBS): 100%, posterior probability (PP): 1]. Moreover, the analysis identified *Bolbitis* and *Elaphoglossum* as sister taxa (MLBS: 100%, PP: 1), which is consistent with previous studies (Moran et al. 2010).

## Conclusions and discussion

In this study, we present the complete chloroplast genome of *Bolbitis angustipinna*, with a total length of 152,562 bp. The genome consists of an LSC, an SSC, and two IRa and IRb regions. Functional annotation revealed 115 genes, including 72 protein-coding genes, 35 tRNA genes, and 8 rRNA genes, with 12 duplicated genes. This genome shows a gene content and structure similar to that of related species.

The phylogenetic analysis of *B. angustipinna* revealed its closest relationship to *B. deltigera*, with strong support from both maximum-likelihood bootstrap values (MLBS: 100%) and posterior probability (PP: 1). This supports the notion that *B. angustipinna* and *B. deltigera* are closely related within the phylogenetic tree. Additionally, our analysis identified *Bolbitis* and *Elaphoglossum* as sister taxa, a relationship that is robustly supported (MLBS: 100%, PP: 1), aligning with previous studies in the field (Moran et al. 2010). The study provides evidence for the systematics of *B. angustipinna* by constructing phylogenetic trees and analyzing their classification positions. Furthermore, the embryonic development pattern of *B. angustipinna* is regarded as a hallmark feature in fern taxonomy and phylogenetic research, which aids in elucidating the evolutionary pathways of related groups within the families Bolbitidaceae and Dryopteridaceae.

In summary, we have constructed the complete chloroplast genome sequence and phylogenetic tree of *Bolbitis angustipinna*, revealing its genetic characteristics and providing further insights into the phylogenetic relationships among populations of this plant species. This work offers data support for future fern species conservation efforts.

## Supplementary Material

Supplemental Material.pdf

## Data Availability

The data of this study are available in GenBank of NCBI at https://www.ncbi.nlm.nih.gov under accession no. PP471972. The associated BioProject, SRA, and Bio-Sample numbers are PRJNA1179923, SRR31166580, and SAMN44509260, respectively.
