# Supplementary material for: The complete chloroplast genome sequence of Bolbitis angustipinna (Hayata) H. Ito (Dryopteridaceae)
Source: Mitochondrial DNA B Resour. 2025 Nov 12;10(12):1159–63. doi: 10.1080/23802359.2025.2582543 (PMC12613301; doi:10.1080/23802359.2025.2582543)
Supplement: Supplemental Material.pdf [file TMDN_A_2582543_SM7714.pdf]

Supplemental Material

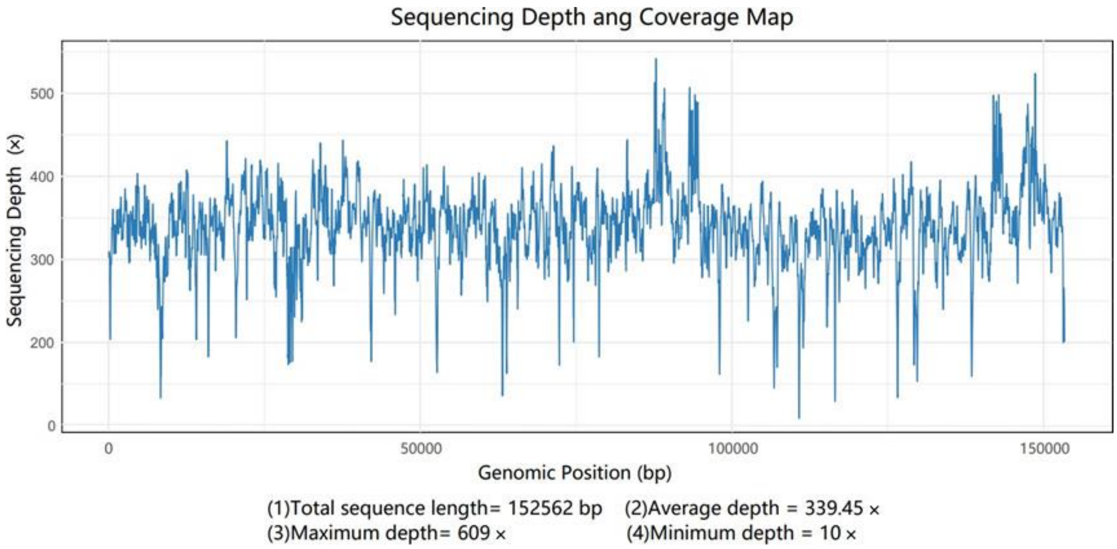

Figure S1. The graph presents the distribution of chloroplast genome sequencing depth for *Bolbitis angustipinna*, with the x-axis representing genomic position and the y-axis corresponding to sequencing depth.

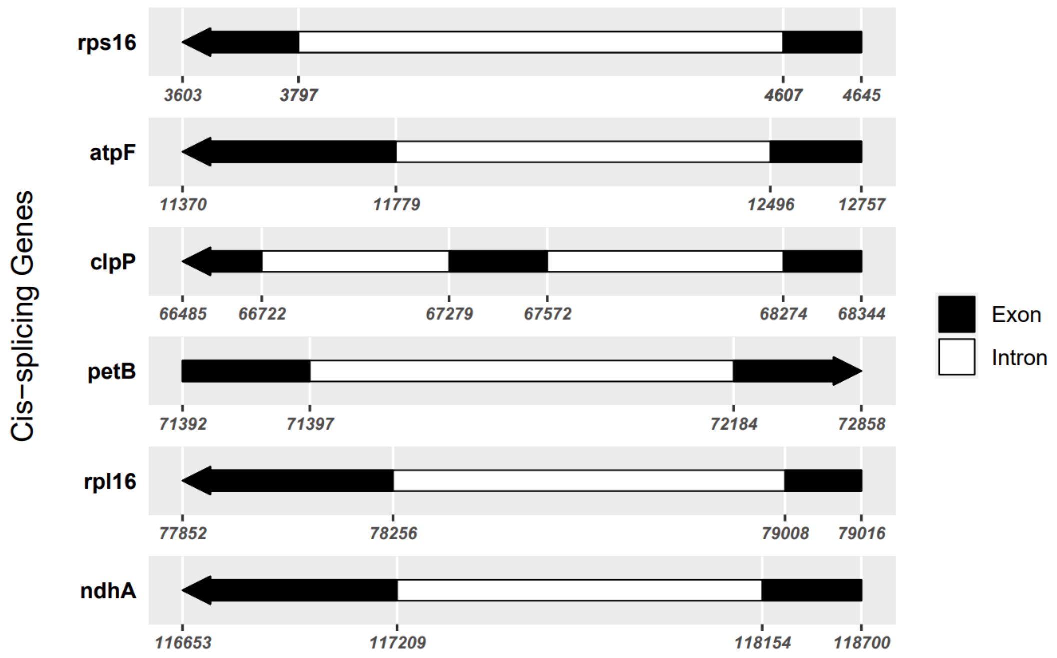

Figure S2. The cis-splicing genes within the chloroplast genome of Bolbitis angustipinna are illustrated in a schematic map generated with CPGView. The exons of these genes are depicted in black, while the introns are shown in white. The direction of transcription is indicated by an arrow. It is important to note that the lengths of the exons and introns are not represented to scale.

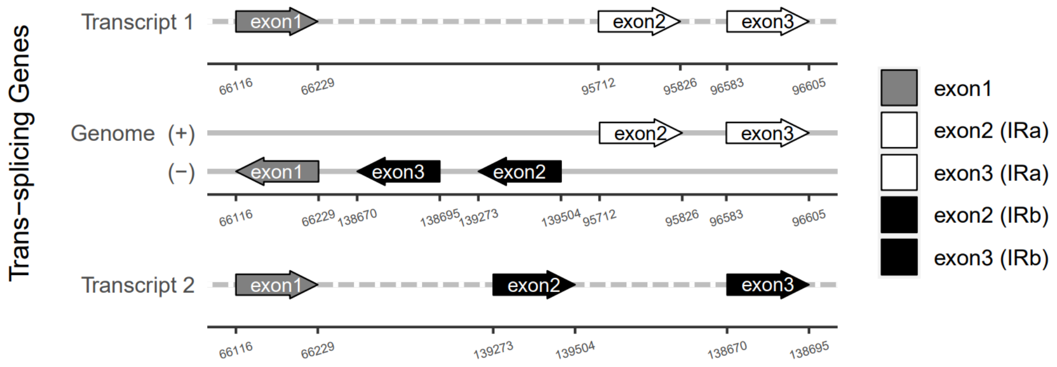

Figure S3. The trans-splicing gene *rps12* in the chloroplast genome of Bolbitis angustipinna is illustrated in a schematic diagram generated using CPGView. This gene comprises three unique exons. The direction of transcription is indicated by an arrow. It is important to note that the lengths of the exons are not represented to scale.
